# Soybean cultivation in lowlands and highlands: production parameters, quality and technological properties of proteins

**DOI:** 10.1002/jsfa.70045

**Published:** 2025-07-16

**Authors:** Patrick da Silva Silva, Janaína Vilella Goveia, Paulo Alberto Azevedo, Rosana Colussi, Nathan Levien Vanier, Moacir Cardoso Elias

**Affiliations:** ^1^ Department of Agroindustrial Science and Technology Faculty of Agronomy Eliseu Maciel, Federal University of Pelotas Capão do Leão Brazil; ^2^ Center for Pharmaceutical and Food Chemical Sciences Federal University of Pelotas Pelotas Brazil

**Keywords:** soy protein, concentrates, absorption, stability

## Abstract

**BACKGROUND:**

Soybean is a legume with high nutritional and functional value, rich in proteins, lipids and vitamins. Protein concentrates represent promising alternatives to animal proteins and their technological properties can be affected by the cultivation system. This study aimed to evaluate the effects of two distinct cultivation systems and environments – lowland and highland soils – on the agronomic performance and quality parameters of grains and their protein concentrates across three soybean genotypes.

**RESULTS:**

Differences were observed among genotypes regarding plant height, with no significant influence of the cultivation system at stage V6; however, at stage R4, variations were noted. The chemical composition varied among genotypes, with BRS 257 exhibiting the highest protein content (428.4 g kg^−1^) in lowland soils. Productivity was higher in two genotypes cultivated in highland soils. On average, the cooking time of grains produced in highlands was shorter (66 min) compared to those produced in lowlands (75 min). In concentrates with at least 800.0 g kg^−1^ protein, the oil absorption capacity and emulsion behavior were not influenced by the cultivation system; however, water absorption was higher (6640 g kg^−1^) in genotypes grown in lowlands. Foam formation and stability in soybeans produced in lowlands showed the highest volumes, which decreased over time but remained superior, except for BMX Delta IPRO.

**CONCLUSIONS:**

The genotypes demonstrated satisfactory agronomic performance, exhibiting adequate potential for both crop ecosystems and promising technological properties for industrial applications, with adaptability to both systems. © 2025 The Author(s). *Journal of the Science of Food and Agriculture* published by John Wiley & Sons Ltd on behalf of Society of Chemical Industry.

## INTRODUCTION

Soybean (*Glycine max* (L.) Merr.), a vegetable consumed in various parts of the world, is rich in high‐quality proteins. It is important not only for integrating vegetarian diets but also for its great nutritional value, excellent functionality and physiological activity, making it a low‐cost protein source. With almost 40% protein and 20% lipids (dry basis), soybeans are an excellent source of oil and protein extraction and are rich in vitamins, dietary fiber, minerals, essential amino acids and isoflavones.^1^ The non‐oily part is widely used in animal feed.

In the south of Brazil, where cultivation began a century ago in the highlands, crop rotation intensified, and soybeans were also included in the lowlands, traditionally used for growing irrigated rice. Areas with floodplain soils, especially in Rio Grande do Sul and Santa Catarina, are opening up to new agricultural alternatives. Integrated rice–soybean–beef cattle systems are being implemented, which offer benefits such as increased availability of mineral nitrogen, enhanced activity of extracellular enzymes and greater microbial diversity, all of which contribute to improved soil quality and the sustainability of agricultural production.^2^

Introducing soybean cultivation into lowland areas presents challenges due to soil characteristics such as low friability and excess water, which can hinder nutrient absorption. Consequently, these factors may impact the crop's quality – an essential aspect for its commercialization and processing.^3^ Studies report that environment and genotype influence grain protein content. This interaction may significantly impact protein concentration and amino acid composition when different locations and cultivation systems are evaluated.^4^

Protein concentrates obtained from cereal or legume grains serve as alternatives or complements to animal‐based protein sources such as milk, meat and eggs, with their growing global demand driving increased production.^5^ Their chemical and structural characteristics can be influenced by the plant species, the cultivar and the cultivation, harvesting and postharvest practices. Depending on the plant source and the extraction method, their technological properties vary, affecting, for example, foaming capacity, water/oil retention capacity, emulsifying ability and solubility.^6^

There are still gaps regarding the impact of cropping systems on the technological quality of grains and their protein concentrates, particularly concerning the integration of agronomic parameters, technological aspects and functional properties in a single study. Addressing these gaps will facilitate improved agronomic and technological utilization of soybeans across different systems and enhance the application of protein concentrates with higher industrial value in food products. The study reported here evaluated three soybean genotypes cultivated under two different cropping systems: lowland soil (planosol) with flat topography and highland soil with undulating topography. It assessed production parameters, technological properties of the grains and the protein concentrates derived from them.

## MATERIALS AND METHODS

### Experimental materials

#### Agronomic characteristics of cultivars

Three cultivars recommended for southern Brazil were selected based on recommendations from their breeders. The cultivar BMX Delta IPRO, whose holder is Brasmax, has an indeterminate growth habit, a cycle of 130 days, a maturation group of 5.9 and a high branching index. It is recommended for cultivation in lowlands and highlands, being resistant to diseases such as stem cancer, bacterial pustule and root rot. The cultivar TecIRGA 6070, whose breeder is the Instituto Rio Grandense do Arroz (IRGA), is also of indeterminate habit, has an average cycle of 134 days, is tolerant to excess water, maturation group 6.3, presents resistance to spot frog eye, stem cancer, bacterial pustule, phytophthalate and lodging. The cultivar BRS 257, developed by Empresa Brasileira de Pesquisa Agropecuária (EMBRAPA), has a certain growth habit, maturation group 6.7, cycle of 129 days and has resistance to diseases such as frog's eye spot, stem cancer, bacterial pustule, common mosaic and root‐knot nematodes, having special characteristic for human food, with absence of the three lipoxygenases enzymes, allowing one to obtain products with good quality and flavor.

#### Production parameters

The genotypes BMX Delta IPRO, TecIRGA 6070 and BRS 257 were grown in the 2020/21 crop, in distant crops 40 km away, in the same physiographic region, southern Rio Grande do Sul, Brazil. Two cultivation systems were used: (a) in lowland, altitude 18 m, flat topography, called lowland soil, planosol or floodplain; (b) in land of undulating topography, altitude 122 m, called highland soil. In both crop systems, the drainage was adequate, and the rainfall regime throughout the cycle was sufficient for the needs of the plants, not requiring artificial irrigation or additional drainage.

In both crops, the experiment consisted of randomized blocks in plots of 12 m^2^, with four lines and a spacing of 0.45 m. According to the respective soil analysis and the official recommendations of Brazilian research for soybean, 110 g of NPK per plot was fertilized in a formulation composed of monoammonium phosphate (10–12% N and 50–54% P) and commercial potassium chloride (58% KCl). The chemical characterization of upland soils shows the following properties: pH (H_2_O) of 5.1, available phosphorus (P) at 14.2 mg dm^−3^, potassium (K) at 78.0 mg dm^−3^, clay content of 190 g kg^−1^ and organic matter content of 21.0 g kg^−1^. In lowland soils, the pH (H_2_O) is 5.7, available phosphorus (P) is 33.2 mg dm^−3^, potassium (K) is 118.2 mg dm^−3^, clay content is 189 g kg^−1^ and organic matter content is 24 g kg^−1^.

#### Harvest and postharvest

The harvest carried out manually with the moisture of the grains close to 18% was made in two lines per plot, with 2 m each, discarding the borders, the threshing being performed in the production field with a small machine, suitable for agricultural experiments (model LUMA BC‐30). The samples were later transported to the Laboratory of Post‐harvest, Industrialization and Grain Quality (LABGRÃOS) of the Faculty of Agronomy Eliseu Maciel of the Federal University of Pelotas (FAEM‐UFPEL), where the grains were cleaned and dried in a stationary dryer of pilot scale with temperature control of 38 °C, until they reached a humidity of 12%.

### Methods

#### Development of plants and parameters of yield

During the vegetative period, agronomic parameters were evaluated, such as plant height – from the soil level to the top of each plant, measured using an agricultural ruler; and the number of pods – counted on 10 randomly selected plants from each row, excluding border plants. The number of seeds per pod was calculated using Eqn (1). The thousand‐grain weight was determined based on the methodology recommended by the International Seed Testing Association,^7^ which involved separating and counting the seeds using a Sanick ESC 2011 electronic seed counter (Santa Catarina, Brazil), through eight repetitions of 100 individually weighed seeds. The final value was obtained by multiplying the average weight by 10 to calculate the weight of 1000 seeds.

(1) $$\mathrm{No}.\;\text{of grains}\;\mathrm{per}\;\text{plot}=\frac{\text{Total grains of each plot}}{\text{Total pods of the plot}}$$

#### Proximal composition

The ash content was determined according to method AOAC 923.03.^8^ A sample was accurately weighed using a balance (Shimadzu AUY220, Japan) in pre‐weighed porcelain crucibles, which were then placed in a muffle furnace (QUIMIS Q318M21, São Paulo, Brazil) at 600 °C for 6 h. The crucible was then removed from the furnace, cooled in a desiccator and weighed. The calculation used is shown in Eqn (2):

(2) $$\mathrm{Ash}\;\left(g\;{\mathrm{kg}}^{-1}\right)=\frac{\mathrm{Ash}\;\text{weight}\;\left(g\right)}{\text{Sample weight}\;\left(g\right)}$$

Protein content was determined according to method 979.09 described by the AOAC.^8^ An acid digestion was first carried out using sulfuric acid and a catalytic mixture composed of sodium sulfate and copper sulfate (7:1). This digestion process took place under intense heating in a Kjeldatherm C mono‐block digestion system (Gerhardt GmbH & Co. KG, Germany) at 380 °C, until the solution became clear, indicating complete digestion of the organic material. After this step, the sample was cooled and the distillation process began using an SL‐74 nitrogen distiller (SOLAB, Piracicaba/SP, Brazil). At this point, 50% sodium hydroxide was added to the sample to convert the ammonium ion into ammonia (NH₃), which was then captured in a 2% boric acid solution. Finally, the captured ammonia was quantified by titration with a 0.1 mol L^−1^ hydrochloric acid solution and three drops of mixed indicator solution (methyl red and bromocresol green). The calculation is shown in Eqn (3):

(3) $$\text{Crude protein}\;\left(g\;{\mathrm{kg}}^{-1}\right)=\frac{100\times 0.014\times 6.25\times \left(V_{a}-V_{b}\right)\times F\times N}{\text{Sample weight}\;\left(g\right)}$$

where *V*_a_ = volume of 0.1 mol L^−1^ hydrochloric acid used in the titration; *V*_b_ = volume of 0.1 mol L^−1^ hydrochloric acid used in the blank test; *F* = correction factor of the 0.1 mol L^−1^ hydrochloric acid solution; *N* = normality of the hydrochloric acid = 0.1; 6.25 = nitrogen‐to‐protein conversion factor.

Lipid content was determined according to method 920.39 described by the AOAC.^8^ A Soxhlet lipid extractor (Lucadema 145/6, São Paulo, Brazil) was used. A dried sample was weighed inside a filter paper cartridge and placed in the extractor, which was attached to the Soxhlet apparatus. Flat‐bottom flasks were previously cleaned, weighed and filled with hexane solvent for an 8 h reflux in the equipment. Afterward, the flasks were placed in an oven to evaporate any remaining solvent, then cooled in a desiccator and subsequently weighed. The calculation is shown in Eqn (4):

(4) $$\text{Lipids}\;\left(g\;{\mathrm{kg}}^{-1}\right)=\frac{\text{Flask weight}+\text{lipids}\;\left(g\right)-\text{empty flask weight}\;\left(g\right)}{\text{Initial weight}\;\left(g\right)}$$

Moisture content was determined according to method 352.2 described by ASAE.^9^ The analysis was performed using accurately weighed samples placed in a container of known weight. The samples were then placed in an oven (QUIMIS Q317M‐22, São Paulo, Brazil) at 105 °C until a constant weight was achieved. After reaching constant weight, sample and container were cooled in a desiccator and weighed. The calculation performed is shown in Eqn (5):

(5) $$\text{Moisture}\;\left(g\;{\mathrm{kg}}^{-1}\right)=\frac{\text{Initial weight}\;\left(g\right)-\text{final weight}\;\left(g\right)}{\text{Initial weight of the sample}\;\left(g\right)}$$

The total carbohydrate content was calculated by difference, based on the values of the other components, as previously described^10^:

(6) $$\text{Total carbohydrates}\left(g\;{\mathrm{kg}}^{-1}\right)=1000–\left(\mathrm{ash}+\text{crude protein}+\text{lipids}+\text{moisture}\right)$$

#### Cooking time

The grains were hydrated overnight before cooking, with the time evaluated in a Mattson cooker.^11^ The equipment, composed of 25 vertical rods each weighing 90 g, was placed in a 2000 mL beaker containing 400 mL of boiling distilled water, kept on an electric plate (Fisatom 752A, São Paulo, Brazil) with controlled heating. Each rod was supported on a grain during the cooking period. The time was started when the water reached 90 °C, and the cooking time was considered as the moment when the 13th rod fell, indicating that more than 50% of the grains were cooked.

#### Soybean protein concentrates

The protein concentrates were produced according to a methodology described previously.^12^ Grains that had been previously ground in a domestic coffee grinder and defatted using hexane for 8 h in a Soxhlet apparatus were further processed in a Perten 3100 mill (Perten Instruments, Huddinge, Sweden). The resulting particles were standardized to a uniform size using a 35‐mesh sieve. The resulting flour was immersed in water at a ratio of 10:1 (water:flour), with the pH adjusted to 8.0 using 2.0 × 10^−9^ mol L^−1^ NaOH, and the mixture was stirred on a magnetic stirrer (Fisatom 752A) for 2 h at room temperature. It was then centrifuged in a centrifuge (Kasvi K14‐4005, Curitiba, Brazil) at 10 000 × *g* for 15 min at 4 °C. The supernatant was adjusted to pH 4.5 using 1.0 × 10^−9^ mol L^−1^ HCl, and after 2 h at 4 °C, a second centrifugation was performed at 10 000 × *g* for 20 min. The precipitate was then redispersed in distilled water and neutralized to pH 7.0 with 2.0 × 10^−9^ mol L^−1^ NaOH. The samples were kept at −80 °C until freeze drying.

##### Electrophoresis in SDS‐PAGE

The electrophoretic profile was evaluated using 5 mg of protein concentrate dissolved in 1 mL of buffer solution containing Tris–HCl (0.5 mol L^−1^, pH 6.8), 10% sodium dodecylsulfate (SDS), 10% glycerol, 5% *β*‐mercaptoethanol and 0.1% bromophenol blue.^13^ The mixture was heated to 90 °C during 5 min. A 5 μL aliquot was added to the gel (4% stacking; 8–16% separation). The buffer used was Tris–glycine SDS, and the protein pattern was 6.5–66 kDa (Sigma‐Aldrich, St Louis, MO, USA) for molecular weight marking, with subsequent staining with Coomassie Blue.

##### Water absorption capacity and oil absorption capacity

Water absorption capacity was determined according to previously described protocols.^14^ Briefly, a 100 mg protein concentrate sample was mixed with 1000 μL of distilled water and stirred in a vortex (Kasvi K45‐2810, Curitiba, Brazil) shaker for 1 min. The suspension was centrifuged at 1800 × *g* for 20 min at 22 °C, and the tube was drained at a 45° angle for 10 min. The absorption capacity was calculated using Eqn (7):

(7) $$\text{Water absorption capacity}\;\left(g\;{\mathrm{kg}}^{-1}\right)=\frac{\text{Volume of water absorbed}\;\left(\mathrm{mL}\right)}{\text{Concentrate sample}\;\left(g\right)}$$

The oil absorption capacity was determined according to previously described protocols.^15^ Briefly, 100 mg of protein concentrate was mixed with 1000 μL of sunflower oil for 30 s. The emulsion remained at room temperature (20 °C) for 30 min and then was centrifuged at 13 600 × *g* for 10 min at 25 °C. The tube was drained at an angle of 45 °C for 20 min. The oil absorption capacity was calculated using Eqn (8):

(8) $$\text{Oil absorption capacity}\left(g\;{\mathrm{kg}}^{-1}\right)=\frac{\text{Volume of oil absorbed}\left(\mathrm{mL}\right)}{\text{Weight of the concentrate sample}\left(g\right)}$$

##### Colorimetric profile

The colorimetric profile was determined using a colorimeter (Minolta, model CR‐310, Osaka, Japan). The color readings were performed in a three‐dimensional system, in three axes. Axis L* represents the value for luminosity and evaluates the sample from white (+) to black (−); axis a* represents the value from red (+) to green (−); and axis b* represents the value from yellow (+) to blue (−).

##### Emulsion stability

To analyze the emulsion stability, dispersions of 15 mL of a 1% solution of protein concentrate were homogenized with 5 mL of corn oil at 12 000 rpm for 2 min (Ultra Turrax, PT 3100, Polytron, Switzerland). The tubes were kept in the dark without agitation, at room temperature, and sealed. The stability monitoring was done visually during 9 days of storage with daily photographs from day 1 to day 9.^16^

##### Formation and stability of foam

Formation and stability of foam were evaluated using a previously described method, with adaptations.^17^ A total of 0.5 g of protein concentrate was diluted in 25 mL of distilled water in a 100 mL beaker, with subsequent stirring at 10 000 rpm for 60 s (Ultra‐Turrax T25, IKA Ltd, Germany). Foam formation was determined by the increase in volume (mL) in relation to the initial volume (protein concentrate + water), and the stability was evaluated by measuring the volume after 30, 60, 120 and 180 min.

#### Statistical analysis

The data were submitted to an analysis of variance in 5% probability by the Tukey test in Statistica® 6.0 software. Principal component analysis (PCA) performed multivariate analysis, with the results presented in graphical form.

## RESULTS AND DISCUSSION

### Development of plants and production yield parameters

There was no statistical difference between the cultivation systems or between the genotypes in the evaluation at 30 days of cultivation (Table 1). At 60 days, no significant differences were observed among the genotypes cultivated in the highland area. However, in the lowland area, the Tec IRGA 6070 genotype (112.5 cm) was taller than both BMX Delta IPRO and BRS 257. In the lowland system, the Tec IRGA 6070 and BRS 257 genotypes reached greater heights. The greater growth in lowland areas may be related to the fact that these soils retain more moisture, which favors plant growth. Additionally, the plants are less exposed to strong winds due to the terrain's topography, which can hinder growth and/or increase water loss through evapotranspiration.

**Table 1:** Plant height at 30 days (V6) and 60 days (R4) of the three soybean genotypes cultivated in highland and lowland cultivation systems

Genotype	30 days (V6)		60 days (R4)	
	Cultivation systems			
	Highland	Lowland	Highland	Lowland
BMX delta IPRO	32.83 ± 3.39 aA	34.37 ± 3.02 aA	87.02 ± 4.17 aA	91.18 ± 3.70 bA
Tec IRGA 6070	37.56 ± 6.21 aA	40.0 ± 4.89 aA	79.25 ± 11.42 aB	112.5 ± 1.29 aA
BRS 257	35.41 ± 6.16 aA	38.62 ± 1.45 aA	78.18 ± 5.62 aB	96.12 ± 9.07 bA
CV (%)	12.29		7.50	

Means followed by different lowercase letters in a column and uppercase in a row in the comparisons between genotypes and cultivation systems indicate significant differences (*P* < 0.05) by the Tukey test. V6, sixth leaf node formed and fifth trifoliolate leaf; R4, a formed pod located in the last four nodes of the main stem; CV, coefficient of variation.

The values found (Table 1) are compatible with those reported by Kambhampati *et al*.,^18^ who evaluated 27 soybean cultivars (R4 and R5) and obtained values between 71 and 108 cm. Giacomeli *et al*.^19^ reported plant heights in stage R5 of 99.19 cm, which are consistent with those of the present study. Plant height is an important characteristic because it is associated with lodging and may affect grain yield and quality.^20^

The number of pods per plant (Table 2) did not differ between genotypes in the same cultivation system, but the BMX Delta IPRO genotype presented a higher number of pods per plant in lowland cultivation. Giacomeli *et al*.^19^ evaluated the number of pods per plant in the lowlands and reported averages of 62.68 pods, closer to those found for the BMX Delta IPRO genotype.

**Table 2:** Production parameters of three soybean genotypes produced in two cultivation systems

Cultivation system	Genotypes			
	Parameters	BMX Delta IPRO	Tec IRGA 6070	BRS 257
Highland	No. of pods per plant	40.17 ± 4.82 aB	41.92 ± 15.22 aA	53.33 ± 7.70 aA
Lowland		63.44 ± 1.71 aA	49.83 ± 0.57 aA	49.06 ± 3.84 aA
CV (%)		15.00		
Highland	No. of grains per pod	2.56 ± 0.07 aA	2.29 ± 0.05 bB	2.53 ± 0.05 aA
Lowland		2.53 ± 0.00 aA	2.50 ± 0.07 aA	2.42 ± 0.13 aA
CV (%)		3.10		
Highland	Weight of one thousand grains (g)	151.58 ± 6.18 bA	131.26 ± 2.88 cA	164.08 ± 4.40 aA
Lowland		135.36 ± 3.21 aB	137.70 ± 1.34 aA	129.64 ± 4.89 aB
CV (%)		2.90		
Highland	Yield (kg ha^−1^)	3558.67 ± 22.06 cB	4370.51 ± 54.77 bA	4539.65 ± 10.82 aA
Lowland		4934.96 ± 44.72 aA	4228.10 ± 29.18 bB	3691.38 ± 15.54 cB
CV (%)		0.79		

For each parameter, means followed by different lowercase letters in a row and uppercase letters in a column in the comparisons between genotypes and cultivation systems indicate significant differences (*P* < 0.05) by the Tukey test.

CV, coefficient of variation.

The number of grains per pod (Table 2) in the highland area was lower in the Tec IRGA 6070 genotype, with 2.29. In the lowland system, there were no differences between genotypes. Among the cropping systems, the highland Tec IRGA 6070 had a lower number of grains per pod than the other two genotypes, which did not differ from each other. According to what was previously discussed, the differences reported in the study are related to the phenotypic expression of the plants, highlighting the genotype × environment interaction. Guesser *et al*.^21^ reported an average value of 1.94 in soybeans cultivated in lowlands, lower than those obtained in the present study for both cultivation systems.

The weight of a thousand grains is an important parameter in evaluating grain yield and quality. It is associated with chemical and technological properties and is influenced by the dimensions and the amount of organic and mineral constituents present.^22^ The highland cultivation system produced an average thousand‐grain weight of 148.97 g (Table 2), while the lowland system produced an average of 134.23 g. In the highland area, the thousand‐grain weight was highest in the BRS 257 genotype at 164.08 g, while in the lowland area, there were no significant differences between genotypes for this parameter. The upland system resulted in a higher thousand‐grain weight in at least two genotypes. The BRS 257 genotype was developed by EMBRAPA for human consumption, with a focus on producing larger and more uniform grains, emphasizing grain quality. Therefore, despite the numerical variation in the number of pods produced per plant without statistical difference in the upland system, the plant can express its potential in grain filling, producing heavier grains that are more attractive to the food market. Guesser *et al*.^21^ evaluating soybean yield components in lowland soils, reporting a mean value of 164.0 g for the thousand‐grain weight. This is similar to the values found in the highland areas of the present study but higher than those found for cultivation in lowland areas, likely due to other production factors. Despite the varying moisture contents (Table 3), averaging approximately 123.0 g kg^−1^, this variable did not influence the results obtained.

**Table 3:** Proximal composition and cooking time of grains from three soybean genotypes produced in two cultivation systems

Cultivation system	Genotypes			
	Component (g kg^−1^)	BMX delta IPRO	Tec IRGA 6070	BRS 257
Highland	Proteins	369.2 ± 0.82 cA	383.3 ± 0.63 bA	409.0 ± 0.67 aB
Lowland		378.4 ± 0.22 bA	377.7 ± 0.70 bA	428.4 ± 0.05 aA
CV (%)		1.39		
Highland	Lipids	210.8 ± 0.66 aB	213.0 ± 2.71 aA	189.0 ± 0.25 bB
Lowland		231.0 ± 1.19 aA	204.0 ± 0.94 bA	234.7 ± 0.38 aA
CV (%)		5.90		
Highland	Ash	41.8 ± 0.02 bB	43.9 ± 0.02 bB	53.2 ± 0.04 aA
Lowland		47.1 ± 0.09 aA	45.8 ± 0.02 bA	47.4 ± 0.04 aB
CV (%)		3.75		
Highland	Moisture	124.5 ± 0.07 aA	126.0 ± 0.00 aA	124.5 ± 0.07 aA
Lowland		125.0 ± 0.00 aA	121.5 ± 0.21 bB	120.5 ± 0.07 bB
CV (%)		0.81		
Highland	Carbohydrates^a^	253.6 ± 1.54 aA	233.7 ± 2.06 bA	224.3 ± 0.38 cA
Lowland		218.4 ± 1.02 bB	249.0 ± 0.02 aA	168.9 ± 0.01 cB
CV (%)		5.09		
Highland	Cooking time (min)	76.50 ± 1.27 bA	88.00 ± 1.41 aA	61.20 ± 0.85 cB
Lowland		69.00 ± 1.70 aB	60.04 ± 1.39 bB	69.30 ± 1.27 aA
CV (%)		1,90		

Means followed by different lowercase letters in a row and uppercase letters in a column in the comparisons between genotypes and cultivation systems indicate significant differences (*P* < 0.05) by the Tukey test.

CV, coefficient of variation.

^a^ Total carbohydrates.

Grain yield (Table 2) was higher in the highland area for the Tec IRGA 6070 and BRS 257 cultivars. In the lowland area, the highest yield was from BMX Delta IPRO, with no significant differences among the other genotypes. Higher yields in highlands are reported in the literature,^23^ being attributed to the fact that lowlands have less favorable characteristics to the cultivation of soybean, such as greater drainage difficulty which can affect the metabolism of plants and reduce their average yield. Although different yield levels were observed in our study, the genotypes exhibited competitive values, consistent with those of the literature, particularly those cultivated in lowland areas where lower results are typically expected, yet they demonstrated satisfactory performance and productivity. A study conducted in commercial soybean fields in lowland areas of Rio Grande do Sul reported yields ranging from 2667 to 3290 kg ha^−1^.^24^ The present study showed satisfactory yields in both cropping systems.

### Chemical composition and cooking behavior of grains

Protein contents varied significantly among genotypes and cropping systems (Table 3). The BRS 257 genotype showed the highest protein levels among the genotypes, with 428.4 g kg^−1^ in lowland areas and 409.0 g kg^−1^ in upland areas, which are consistent with the literature. Tec IRGA 6070 showed no variation in this parameter between systems. BMX Delta IPRO presented the lowest protein contents. In line with what has been previously discussed, as a genotype developed for human consumption, BRS 257 underwent genetic selection for superior nutritional quality, particularly regarding protein content, which is one of the main attributes for direct consumption. Whaley and Eskandari,^25^ evaluating two soybean populations under five environmental conditions, found significant effects of genotype × environment interaction for both grain yield and protein concentration.

Literature data report average protein concentrations of 361 g kg^−1^,^26^ which align with the commercial varieties used in the experiment (BMX Delta IPRO and Tec IRGA 6070), but is lower than that of special high‐protein genotypes, which exceed 400.0 g kg^−1^. Epie *et al*.^27^ state that protein concentration is influenced by both genotype and environmental factors.

Lipid contents also showed relevant differences. Among the genotypes, the highest levels were observed in BMX Delta IPRO and Tec IRGA 6070 under upland conditions, with 210.8 and 213.0 g kg^−1^, respectively, with no statistical difference between them. In lowland areas, BMX Delta IPRO and BRS 257 did not differ, presenting higher lipid levels compared to Tec IRGA 6070. It is also noteworthy that BMX Delta IPRO maintained high and stable lipid contents across both systems, demonstrating its consistency in this component.

Junior *et al*.^26^ report average oil concentrations of 221.0 g kg^−1^, consistent with the findings of the present study. Similarly, Baisch *et al*.^28^ document average oil concentrations ranging from 276.0 to 202.0 g kg^−1^ in soybeans cultivated in lowland areas, aligning with the current study's results. Ody *et al*.,^29^ evaluating soybean oil content in lowland areas with early sowing and soil scarification, found values ranging from 115.1 to 229.5 g kg^−1^, highlighting the importance and interaction between sowing times and soil preparation. Soil moisture stress is reported in the literature as one of the factors responsible for variations in oil and protein content,^30^ which helps to explain the variations found in the study. Although rainfall levels were sufficient for crop development, the different environments have particular soil characteristics that may influence results.

In upland cultivation, the BRS 257 genotype presented the highest value (53.2 g kg^−1^) of ash content (Table 3). In lowland areas, the values ranged from 45.8 to 47.4 g kg^−1^. These values are consistent with reports in the literature (48.0 g kg^−1^).^1^ This higher content of ash found in BRS 257 may be related to the greater accumulation of minerals by the plant in upland systems.

Moisture content is described in Table 3. In upland areas, the variations ranged from 124.5 to 126.0 g kg^−1^, with minimal variations and no statistical differences. In lowland areas, the variations were greater, ranging from 120.5 to 125.0 g kg^−1^.

Carbohydrates were lower in both cultivation systems for the BRS 257 cultivar. Since carbohydrates were calculated by difference, without direct measurement, the values are justified as a consequence of the composition of the other constituents, reflecting their sum. Given the higher protein and ash contents, it is expected that the genotype in question would have lower carbohydrate levels. The variation ranged from 224.3 to 253.6 g kg^−1^ in upland areas and from 168.9 to 249.0 g kg^−1^ in lowland areas. The study aimed solely to compare the values between different genotypes produced in two cultivation systems, without applying any specific treatment that could contribute to increases in any fraction of the proximate composition.

Cooking time (Table 3) varied between genotypes and cultivation locations. For the grains produced in upland areas, the longest cooking time was 88.0 min (Tec IRGA 6070), while in lowland areas, BMX Delta IPRO with 69.0 min and BRS 257 with 69.3 min did not differ from each other but were longer than Tec IRGA (60.04 min). In general, genotypes grown in upland areas had longer cooking times than those grown in lowland areas, except for the BRS 257 genotype grains. This behavior may be associated with the higher protein and ash contents described in the proximate composition (Table 3), which, although higher in lowland areas, still maintain competitive performance with the other genotypes. This shorter cooking time found in upland areas reinforces its technological profile, favorable for human consumption. The average cooking time for genotypes produced in upland areas was 75 min, while for those produced in lowland areas, it was 66 min.

The reduction in cooking time from 76.50 to 69.00 min (BMX Delta IPRO) reinforces the genotype's sensitivity to the environment/cultivation system. These results highlight that cooking time is a characteristic influenced by interactions between genotype, environment and chemical composition. Cooking time depends on the chemical composition, which is influenced by genetic and environmental components, planting times and locations and the interaction of these factors.^31^ For direct human consumption, this technological parameter can result in greater or lesser time and cost savings in the process.

Biscaro *et al*.^32^ evaluated possible changes caused by irradiation on cooking time and soaking in the BRS 257 cultivar, finding a significant variation to 33 min after irradiation, compared to the 77.3 min required before irradiation, similar to those found in the present study among the three genotypes. The various processes to which soybeans are subjected for consumption include roasting, soaking, boiling, steaming and/or fermentation. These treatments can improve characteristics such as flavor, palatability and bioavailability of bioactives, and even deactivate antinutritional compounds.^33^

Although it is not the focus of the research, there are already reports in the literature indicating that, in addition to the genotype × environment effect, factors such as temperature also influence productivity and the quality of important grain traits, such as protein and oil content.^34^ These parameters are essential for the soybean seed industry and for human/animal nutrition.

### Percentage of protein, characterization and technological properties of protein concentrates

The protein contents of all three genotypes produced in both highland and lowland systems exceeded 800.0 g kg^−1^ of crude protein, categorizing them all as protein concentrates (Table 4). According to Kim *et al*.^35^ soy protein concentrates should have a minimum of 65% protein. If they had presented values above 90%, they would have been classified as protein isolates. Advances in the production chain, as well as the introduction of soybean cultivation in lowland areas, should be able to maintain not only grain productivity but also its quality, as soil characteristics influence quality indicators, affecting technological properties, which are of fundamental importance for the destination of the grains, especially when the focus is on human or animal consumption. The production of protein concentrates highlighted the quality of the raw material in both systems, even though they were different environments that provide distinct nutrient absorption and physicochemical characteristics.

**Table 4:** Protein content in concentrates from three soybean genotypes produced in two cultivation systems

Genotype	Degree of purity in protein (g kg^−1^)	
	Highland	Lowland
BMX Delta IPRO	872.0 ± 0.70 aA	800.6 ± 0.20 bB
Tec IRGA 6070	804.4 ± 0.65 bB	835.8 ± 0.12 aA
BRS 257	800.6 ± 0.04 bA	801.9 ± 1.01 bA
CV (%)	0.71	

Means followed by different lowercase letters in a column and uppercase in a row in the comparisons between genotypes and cultivation systems indicate significant differences (*P* < 0.05) by the Tukey test.

CV, coefficient of variation.

**Figure 1:**
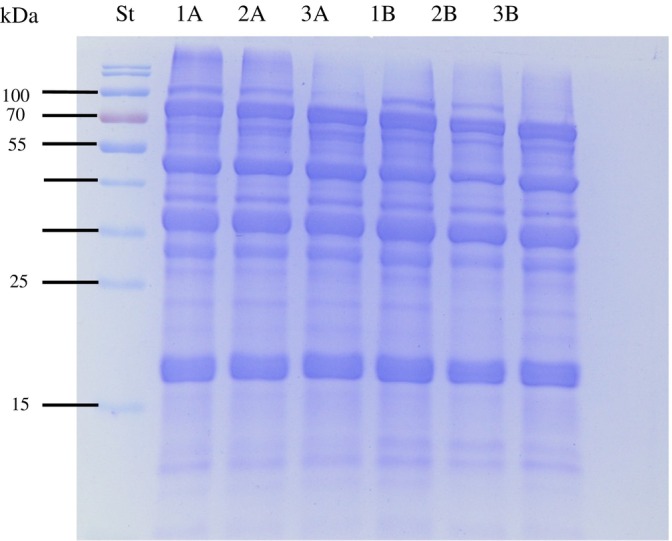
SDS‐PAGE profiles of protein concentrate of three soybean genotypes produced in two cultivation systems. St, standard proteins; 1A, BMX Delta IPRO; 2A, Tec IRGA 6070; 3A, BRS 257 in highland system; 1B, BMX Delta IPRO; 2B, Tec IRGA 6070; 3B, BRS 257 in lowland system.

The observation of protein bands by SDS‐PAGE reveals changes in the patterns of protein concentrates, indicating that protein structure is influenced by both genotype and cultivation system of the soybeans. The genotypes exhibited dissimilarities in bands above 100 kDa, which varied between different cultivation systems (Fig. 1).

The results of this study align with those reported by Krishnan *et al*.,^36^ who attributed the concentration of high‐molecular‐weight proteins in soybeans to greater accumulation of specific components of subunits *β*‐conglycinin and glycinin. This is presumably influenced by the preferential expression of respective genes during grain development. As soil and insolation conditions in highland and lowland crop systems are different, these characteristics probably influenced each genotype differently.

**Figure 2:**
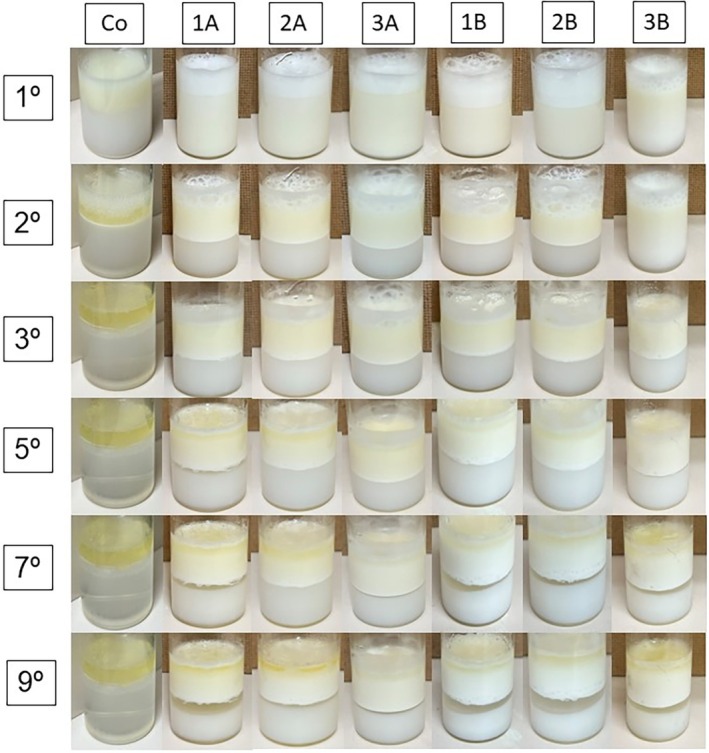
Emulsion capacity and stability for 9 days of protein concentrate obtained from three soybean genotypes produced in two cultivation systems. Co, control (water + oil); 1A, BMX Delta IPRO; 2A, Tec IRGA 6070; 3A, BRS 257 produced in highland system; 1B, BMX Delta IPRO; 2B, Tec IRGA 6070; 3B, BRS 257 produced in lowland cultivation system.

**Figure 3:**
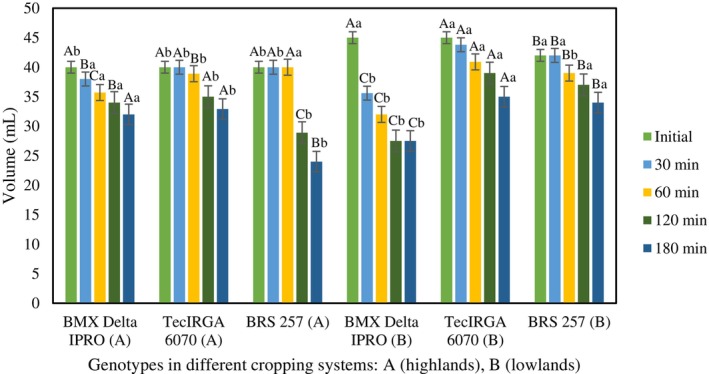
Foaming capacity and stability of protein concentrates over 180 min from three soybean genotypes produced in two cultivation systems. For each parameter, bars with uppercase letters compare genotypes under the same system, and lowercase letters compare cultivation systems.

The protein contents of the grains (Table 3) were affected by the genotype and the cultivation system, and the protein profile (Fig. 1) also showed the same influences, which are reflected in the technological properties of the grains (Table 3) and protein concentrates (Table 4 and Figs 1, 2, 3). These behaviors corroborate the effects of the interaction between genotype and environment.^4^, ^25^

In the highland cultivation system, the Tec IRGA 6070 genotype presented higher water absorption capacity (Table 5), with 5170.0 g kg^−1^, while the other genotypes did not differ from each other. In the lowland system, the three genotypes showed higher water absorption capacity (2580.0 to 6640.0 g kg^−1^) than those cultivated in the highland system. These values are compatible with studies by Lindemann *et al*.,^37^ which report values between 2110.0 and 3360.0 g kg^−1^. The differences found between the genotypes and systems reinforce the importance of considering such factors when selecting raw materials, according to their future application in food industry formulations. Applications in products that require higher water absorption make those produced in lowland areas excellent options, with an emphasis on the Tec IRGA 6070 genotype. This approach positions the present study as one of the pioneers in relating production system factors in upland and lowland areas to functional properties.

**Table 5:** Water and oil absorption capacity of protein concentrates from three soybean genotypes produced in two cultivation systems

Genotype	Water absorption capacity (g kg^−1^)		Oil absorption capacity (g kg^−1^)	
	Highland	Lowland	Highland	Lowland
BMX Delta IPRO	2090.0 ± 0.01 bB	2580.0 ± 0.09 cA	6550.0 ± 0.01 aA	5990.0 ± 0.02 bB
BRS 257	2230.0 ± 0.03 bB	3200.0 ± 0.17 bA	5800.0 ± 0.00 cA	5930.0 ± 0.01 bA
Tec IRGA 6070	5170.0 ± 0.00 aB	6640.0 ± 0.17 aA	6200.0 ± 0.18 bB	6420.0 ± 0.01 aA
CV (%)	2.84		1.24	

For each parameter, means followed by different lowercase letters in a column and uppercase letters in a row in the comparisons between genotypes and cultivation systems indicate significant differences (*P* < 0.05) by the Tukey test.

CV, coefficient of variation.

In the highland production system, the BMX Delta IPRO genotype exhibited the highest oil absorption (6550.0 g kg^−1^), and in the lowland Tec IRGA 6070 (6420.0 g kg^−1^) (Table 5). The highland production system allowed greater oil absorption, except for Tec IRGA 6070. Meganaharshini *et al*.,^5^ evaluating the technological properties of different commercial protein isolates, reported oil absorption values in beans and peas of 6.90 and 7.20 g g^−1^, respectively, which are similar to the values found in this study.

When considering the application of protein concentrates in the production of meat and sausage analogs, the combination of water and oil absorption is essential. Absorbed water influences texture, while absorbed fat intensifies taste satisfaction.^38^ In food formulations in which the characteristic of oil retention is relevant, protein concentrates can serve as an excellent alternative.^39^ Based on the functional properties of water and oil absorption capacity, it is possible to apply them in the food industry, making them viable for different products. The variations between genotypes and environments can be strategically explored, depending on the application, and are considered promising ingredients for food use.

In the colorimetric profile (Table 6), the protein concentrates from genotype Tec IRGA 6070 presented the highest luminosity value (L* = 83.14). In the lowlands, there were no differences between genotypes. The protein concentrates obtained from soybeans produced in the lowlands presented the highest values of L* (81.21–83.14).

**Table 6:** Colorimetric profile of protein concentrates from three soybean genotypes produced in two cultivation systems

Cultivation system	Genotypes			
	Value	BMX Delta IPRO	Tec IRGA 6070	BRS 257
Highland	L*	75.57 ± 2.17 bB	76.25 ± 4.54 bB	80.01 ± 0.87 aA
Lowland		82.43 ± 0.39 aA	83.14 ± 0.40 aA	81.21 ± 0.20 aA
CV (%)		2.63		
Highland	a*	1.00 ± 0.09 aA	0.16 ± 0.08 bB	0.14 ± 0.05 aB
Lowland		0.91 ± 0.13 aA	0.52 ± 0.07 aB	0.25 ± 0.10 aB
CV (%)		33.25		
Highland	b*	14.0 ± 1.03 aA	11.88 ± 0.54 bB	13.48 ± 0.48 bA
Lowland		13.18 ± 0.54 aB	13.74 ± 0.57 aAB	14.43 ± 0.93 aA
CV (%)		5.33		

For each parameter, means followed by different lowercase letters in a row and uppercase letters in a column in the comparisons between genotypes and cultivation systems indicate significant differences (*P* < 0.05) by the Tukey test.

CV, coefficient of variation; L*, lightness; a*, redness; b*, yellowing.

The highest yellow (a*) trends were verified in the protein concentrates of the BMX Delta IPRO genotype of soybeans cultivated in highland (1.00) and lowland (0.91) areas. In the highlands, the Tec IRGA 6070 genotype showed less tendency to red (b* = 11.88), and in the lowlands, BRS 257 presented the highest value (14.43). The results obtained for the colorimetric profile (Table 6) are important for the food industry in defining the incorporation of these proteins into various food products, particularly in formulations analogous to meat, where product color significantly impacts consumer perception.^39^ The approach to the colorimetric profile of protein concentrates aimed to demonstrate the relationship between production systems and factors that affect the composition and structure of proteins, as well as the presence of possible associated compounds. Even though the process for obtaining concentrates is standardized, the initial state of the raw material can influence the results. Despite the significant differences found in the analysis, the values are very close between the genotypes grown under the same system, indicating minimal influence.

The protein concentrates showed emulsifying capacity (Fig. 2). Emulsions exhibited similar behavior until the third day. By the fifth day, phase separation became more noticeable, particularly in concentrates from the BMX Delta IPRO genotype across both cultivation systems. By the seventh day, this separation was more evident in protein concentrates from the Tec IRGA 6070 and BRS 257 genotypes produced in lowland areas compared to highland areas. By the ninth day, all genotypes showed clear phase separation, with a more pronounced effect observed in genotypes produced in lowland areas. These variations are attributed to differences in molecular mass, amino acid profiles and disulfide bonds, which vary depending on genotype and cultivation location, as documented in the literature.^40^ The different characteristics presented by the genotypes as a function of the cultivation environment are evident, being strongly influenced by their behavior over time. Environmental conditions, such as temperature, humidity and nutrient availability, are capable of modifying the molecular characteristics of the grains.

The emulsifying capacity is used to evaluate the emulsifying properties of a protein, expressing how much an emulsion resists changes during a certain period.^41^ This ability can be increased by the action of enzymes, such as transglutaminase,^42^ pepsin, or gum arabic,^43^ maintaining the stability of the emulsions and protecting against oxidation.

Foams are multiphase dispersions of a gas dispersed in a liquid. There is great interest in aerated foods, due to the soft and creamy sensation that they provide in the mouth; thus researchers are studying and understanding the mechanisms of formation and stability of foam.^44^ The stability of foam is responsible for the appearance and structure of foods whose instability can lead to the loss of the fluffy structure and its texture.^45^

The foaming capacity of protein concentrates from soybeans grown in the highland system did not differ (Fig. 3), starting at an initial volume of 40 mL. However, their stability varied among genotypes. BMX Delta IPRO exhibited slow and proportional degradation over time. Tec IRGA 6070 maintained its initial volume for the first 30 min but decreased by 60 min. BRS 257 sustained its volume until 60 min but significantly decreased to less than 25 mL by 120 min.

The behavior of soybean concentrates cultivated in the lowlands (Fig. 3) differed from that of concentrates of the highlands, with higher initial volumes and a greater tendency to instability, which was more pronounced in BMX Delta IPRO, from 45 to 35 mL at 30 min and to 27.5 mL at 120 min. Tec IRGA 6070 and BMX Delta IPRO exhibited similar behaviors, with gradual and slow stability reductions over 180 min.

The protein content of the source is one of the determining factors in the functional properties of protein concentrates.^46^ As presented in Table 4, the genotypes differed between the systems. This is one of the reasons why genotypes grown in lowland areas present higher values for initial foam formation volume – except for BMX Delta IPRO, which, despite having a higher protein content in upland areas, showed the same initial volume as the others.

When it comes to foam formation, not only does protein content influence the outcome, but other factors do as well. Therefore, for the production of protein concentrates, degreased samples were used, as lipids can also interfere. The application in food matrices may have its properties affected, limiting the proteins' ability to form foam, where the use of hydrocolloids – such as gelatin – is recommended to increase stability over a longer period.^5^

These behaviors can be attributed to the dissimilarities observed in the SDS‐PAGE bands (Fig. 1) among the genotypes in the two cultivation systems. Despite the differences, the three genotypes across both systems do not show any compromise in the technological properties of the proteins, but this reinforces the importance of considering the cultivation environment not only for productivity but also for functional/technological properties.

The results (Fig. 3) show that the lowland cultivation system enables greater initial foam formation capacity compared to the upland system. They also demonstrate greater stability over time, except for BMX Delta IPRO, which experienced a drastic reduction in foam volume. Foam formation capacity and stability can be altered by the denaturation of 11S globulin induced by pH changes, potentially resulting in increases of up to 40% compared to the native state.^47^

### Principal component analysis

**Figure 4:**
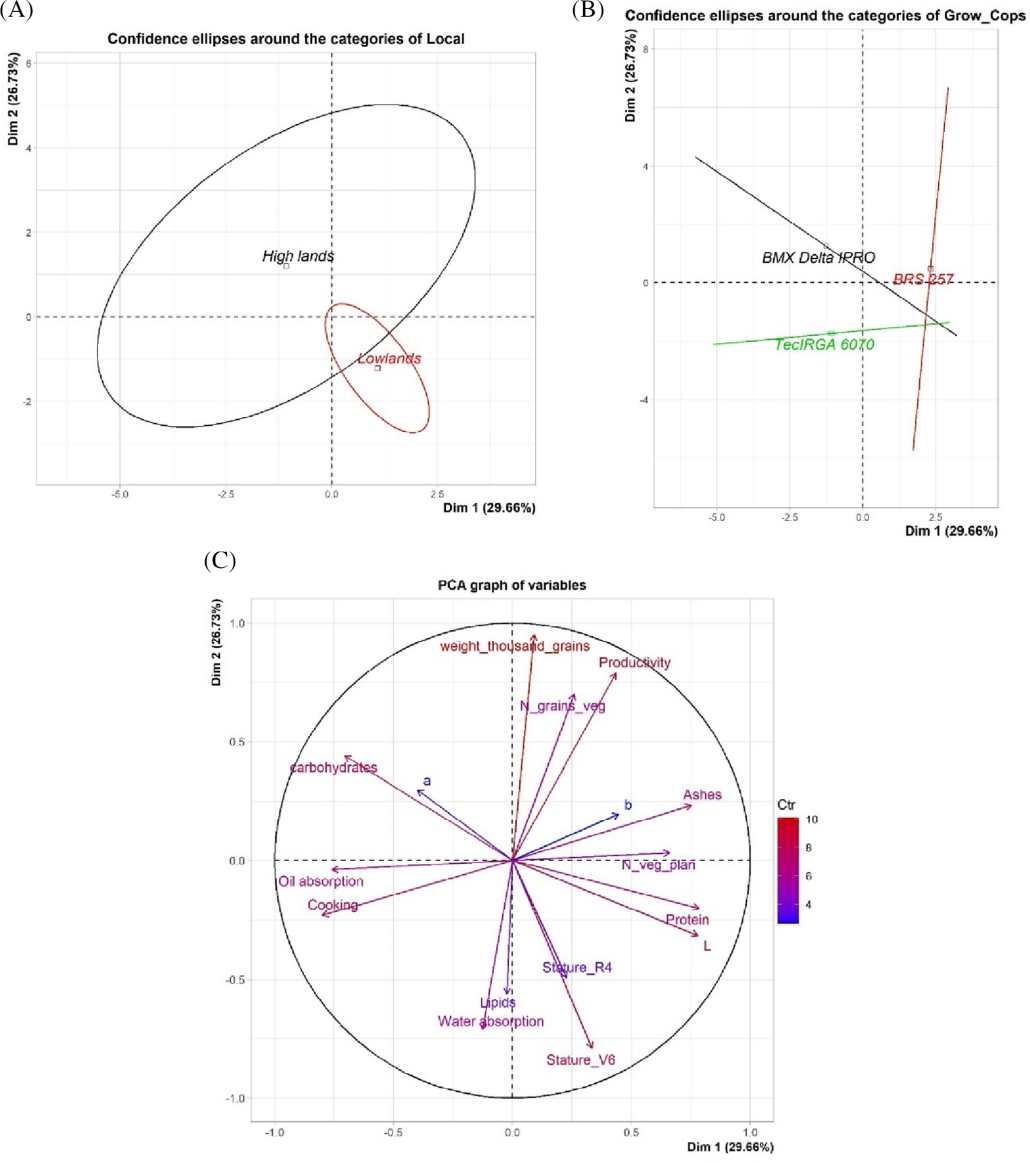
Principal component analysis (PCA), represented by confidence ellipses, demonstrates separation and grouping based on: (A) the different cultivation systems employed; (B) the evaluated soybean genotypes; and (C) the relationships between agronomic production parameters and the technological properties of the grains and their protein concentrates.

Although crop systems present dissimilarities, it is possible to observe a small overlap between the confidence ellipses (Fig. 4(A)). Similar to what was observed in Fig. 2(A), the ellipses overlap (Fig. 4(B)), demonstrating that the three genotypes show similarity to each other. In (Fig. 4(C)) it is possible to observe correlations and overlaps between production parameters and technological properties of proteins. Principal component 1 (Dim 1) explains the largest portion of the data variation, accounting for 29.66%. Principal component 2 (Dim 2) explains the second largest portion of the variation, accounting for 26.73%. There is a clear strong positive correlation between plant height, protein content, number of pods per plant, ash content and the L* value of the colorimetric profile, while these same factors show a negative correlation with carbohydrate content. The positive relationship between plant height and the number of pods per plant occurs because taller plants tend to be more vigorous, with greater biomass accumulation and better development of reproductive structures (pods). Thousand‐seed weight, yield and number of seeds per pod also show a strong positive correlation, which is expected since these are productive traits of the plant.

A negative correlation is observed between cooking time and oil absorption capacity with ash content and productive traits (thousand‐seed weight, number of seeds per pod and yield), as evidenced by the opposing vectors. There is a positive correlation between lipid content and water absorption capacity.

## CONCLUSION

The soybean genotypes BMX Delta IPRO, Tec IRGA 6070 and BRS 257, cultivated in upland and lowland areas of southern Brazil, demonstrated adequate agronomic potential and satisfactory grain quality. This was evident across various parameters, including plant development, yield and chemical composition, indicating their adaptability to diverse ecosystems. While productivity was higher in upland areas, grains from lowland cultivation cooked more quickly. Regardless of the cultivation system or genotype, the electrophoretic profiles and technological properties of the soybeans confirmed the feasibility of producing protein concentrates with a minimum purity of 80% and comparable emulsifying capacities. Notably, protein concentrates from soybeans grown in lowland areas exhibited enhanced water and oil absorption capacities, as well as superior foam formation and stability. Despite variations in technological properties, all three genotypes – whether cultivated in upland or lowland areas – proved viable for producing protein concentrates suitable for various sectors of the food industry, including meat analog products, plant‐based beverages and other soy protein‐based formulations.

## AUTHOR CONTRIBUTIONS

PSS performed field experiments, laboratory analysis and wrote the manuscript; JVG assisted in field experiments; PAA participated in analysis and writing; RC participated in writing; NLV and MCE were responsible for the conception of the idea of the work and for the supervision of field and laboratory experiments.

## CONFLICT OF INTEREST

There is no conflict of interest in the submission of the manuscript and the manuscript was approved by the authors for publication.

## Data Availability

The data that support the findings of this study are available on request from the corresponding author. The data are not publicly available due to privacy or ethical restrictions.
